# Survival Estimation, Prognostic Factors Evaluation, and Prognostic Prediction Nomogram Construction of Breast Cancer Patients with Bone Metastasis in the Department of Bone and Soft Tissue Tumor: A Single Center Experience of 8 Years in Tianjin, China

**DOI:** 10.1155/2022/7140884

**Published:** 2022-01-31

**Authors:** Yao Xu, Haixiao Wu, Guijun Xu, Zhuming Yin, Xin Wang, Vladimir P. Chekhonin, Karl Peltzer, Shu Li, Huiyang Li, Jin Zhang, Wenjuan Ma, Chao Zhang

**Affiliations:** ^1^Tianjin Medical University Cancer Institute and Hospital, National Clinical Research Center for Cancer, Key Laboratory of Cancer Prevention and Therapy, Tianjin's Clinical Research Center for Cancer, Tianjin, China; ^2^The Sino-Russian Joint Research Center for Bone Metastasis in Malignant Tumor, Tianjin, China; ^3^Department of Orthopedics, Tianjin Hospital, Tianjin University, Tianjin, China; ^4^Department of Health Management Center (Epidemiology and Biostatistics), First Affiliated Hospital, Army Medical University, Chongqing, China; ^5^Department of Basic and Applied Neurobiology, Federal Medical Research Center for Psychiatry and Narcology, Moscow, Russia; ^6^Department of Psychology, University of the Free State, Turfloop, South Africa

## Abstract

**Purpose:**

Bone metastasis in breast cancer remains globally concerned. Accurate survival estimation would be beneficial for clinical decision-making, especially for the patients with potential indications of surgery. Based on a retrospective cohort from China, the study aimed to construct a prognostic prediction nomogram for breast cancer patients with bone metastasis.

**Methods:**

Breast cancer patients with bone metastasis diagnosed between 2009 and 2017 in our department were retrospectively selected. The total cohort was divided into construction and validation cohorts (ratio 7 : 3). A nomogram was constructed to predict the probability of survival, and the performance of model was validated.

**Results:**

A total of 343 patients were enrolled with 243 and 100 patients in construction and validation cohorts, respectively. The median overall survival for the total cohort was 63.2 (95% CI: 52.4–74.0) months. Elevated ALP (HR = 1.71, 95% CI: 1.16–2.51; *P*=0.006), no surgery for breast cancer (HR = 2.19, 95% CI: 1.30–3.70; *P*=0.003), synchronous bone metastasis (HR = 1.98, 95% CI: 1.22–3.22; *P*=0.006), and liver metastasis (HR = 1.68, 95% CI: 1.20–2.37; *P*=0.003) were independent prognostic factors for worse survival. The independent predictors and other five factors (including age at diagnosis, ER status, PR status, Her-2 status, and the performance of bisphosphonate) were incorporated to construct the nomogram. The C-index was 0.714 (95% CI: 0.636–0.792) and 0.705 (95% CI: 0.705) in the construction cohort and validation cohort, respectively. All the calibration curves were close to the 45-degree line, which indicated satisfactory calibration.

**Conclusion:**

A retrospective study aiming at prognostic estimation of breast cancer patients with bone metastasis was designed. Four independent prognostic factors were identified and a prognostic nomogram was constructed with satisfactory discrimination and calibration. The model could be used in survival estimation and individualized treatment planning.

## 1. Introduction

Breast cancer (BC) is the most common primary malignant tumor in female. In 2021, there will be 281,550 estimated new cases and 43, 600 estimated deaths in the United States [1]. Due to the developed early detection and comprehensive treatment strategies, the prognosis of BC has been improved in recent years [2, 3]. Despite of the improved prognosis of patients at early stage, metastatic breast cancer (MBC) attributed to the main cause of death among BC patients and it was reported that the 5-year breast cancer-specific survival (BCSS) was dismal 26% [4]. Compared with other MBC patients, the survival of patients with breast cancer bone metastasis (BCBM) was better. The median survival of patients with bone-only metastasis was up to 24–54 months [5, 6]. Due to the high heterogeneity of BC, identification of prognostic factors and prediction of survival time was the prerequisite for individual decision-making.

Except the Department of Breast Cancer, bone metastasis in breast cancer was a common disease in the Department of Bone and Soft Tissue Tumor. Such patients usually chose the Department of Bone and Soft Tissue Tumor for skeletal-related events (SREs) and/or motor dysfunction. Compared with other departments, the patients with synchronous bone metastasis (SBM) are more common. The patients usually require the surgical intervention to release the symptom. Prognostic estimation was one of the most important issues before surgery performance.

Several prognostic factors of BCBM patients have been investigated in previous studies. A retrospective study on 238 cases concluded that breast subtype was associated with overall survival (OS), bone disease-free survival, and survival with bone disease [7]. The 5-year survival rate was up to 40% for luminal A, luminal B, and basal patients, while it was 4% for triple negative breast cancer patients [7]. Another study based on Surveillance, Epidemiology, and End Results (SEER) dataset attested that breast subtype, age at diagnosis, race, tumor grade, and the presence of organ metastasis were independent prognostic factors of BCBM patients [8]. Sufficient literatures on prognostic factors were reported, which was fundamental for subsequent study on survival estimation [9–12].

Clinical prediction models, usually incorporating patients' demographic and clinical characteristics, were an efficient tool to evaluate the probability of disease development and survival outcome [13, 14]. As one of the most widespread predictive models, nomogram was widely reported in several types of cancers [15–17]. However, most prognostic nomograms among MBC patients were constructed based on SEER database [18, 19]. The generalizability in Chinese population remained unclear. Thus, the present study aimed to construct a prognostic prediction nomogram based on the cohort from the Department of Bone and Soft Tissue Tumor, Tianjin Medical University Cancer Institute and Hospital, Tianjin, China. The nomogram can help oncologists estimate prognosis accurately and guide the individualize treatment for Chinese patients.

## 2. Materials and Methods

### 2.1. Data Source and Cohort Selection

**Figure 1:**
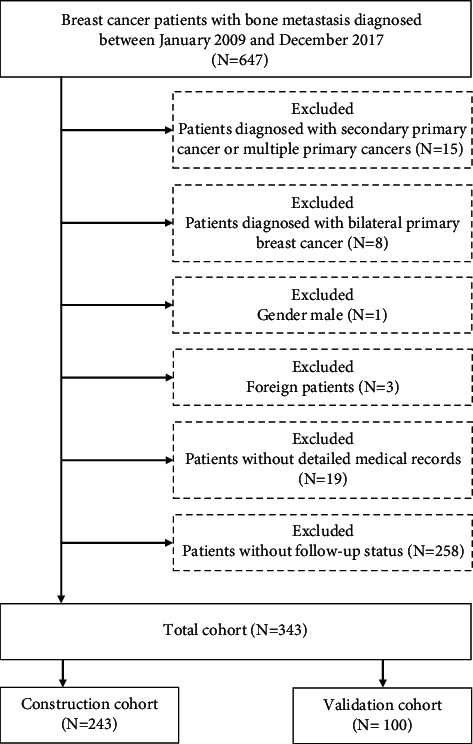
The flowchart of the patient selection.

The medical records of breast cancer bone metastasis (BCBM) patients in the Department of Bone and Soft Tissue Tumors, Tianjin Medical University Cancer Institute and Hospital, were retrospectively collected. All of patients were older than 18 and diagnosed between January 2009 and December 2017. The exclusion criteria were as follows: (1) patients diagnosed with secondary primary cancer or multiple primary cancers; (2) patients diagnosed with bilateral primary breast cancer; (3) male patients; (4) foreign patients; (5) patients without detailed medical records; (6) patients without follow-up status; (7) patients with metabolic bone disease; and (8) patients with severe osteoporosis. The flowchart of the patient selection is listed in Figure 1.

### 2.2. Demographic and Clinical Variables

Patients' demographic and clinical characteristics were included as follows: age at diagnosis (18–45 years, 46–55 years, or >55 years), marital status (married or unmarried), history of smoking (yes or no), alcohol consumption (yes or no), menstrual status (menstruation or menopause), history of abortion (no or yes), family history of cancer (yes or no), histological type (ductal carcinoma or others), tumor grade (Grade I-II or Grade III), tumor size (<2 cm, 2–5 cm, or >5 cm), lymph node metastasis (yes or no), ER status (positive or negative), PR status (positive or negative), Her-2 status (positive or negative), Ki-67 status (positive or negative), the performance of surgery (yes or no), chemotherapy (yes or no), radiotherapy (yes or no), endocrinotherapy (yes or no), and targeted therapy (yes or no). The chemotherapy regimens included AC, CAF, and TAC in the present study.

Laboratory data were investigated and grouped according to normal threshold value in our hospital, including hemoglobin (HGB: 115–150 g/L, <115 g/L, or >150 g/L), carbohydrate antigen 153 (CA153: 0–25 U/ml or >25 U/ml), carcinoembryonic antigen (CEA: 0–5 ng/ml or >5 ng/ml), alkaline phosphatase (ALP: 50–135 U/L, <50 U/L, or >135 U/L), and serum calcium (2.10–2.55 mmol/L, <2.10 mmol/L, or >2.55 mmol/L). ABO blood type was also investigated, being grouped into A type, B type, AB type, and O type.

The records on organ metastasis, skeletal-related events (SREs), and associated treatment were included: the radiotherapy for bone metastatic site (no or yes), pathological fracture (no or yes), spinal cord compression (no or yes), surgery for bone metastatic site (no or yes), performance of bisphosphonate therapy (no or yes), liver metastasis (no or yes), brain metastasis (no or yes), lung metastasis (no or yes), and other organs metastasis (no or yes). As in previous study, synchronous bone metastasis (SBM) was defined as BM diagnosis within 6 months after BC diagnosis, while metachronous bone metastasis (MBM) was defined as BM diagnosis more than 6 months after BC diagnosis [20, 21].

### 2.3. Statistical Analysis

All missing values in the current study were completed by multiple imputation methods. The total cohort was randomly divided into the construction and validation cohort (ratio 7 : 3). The construction cohort was used to identify the prognostic factors for BCBM patients and to construct the predictive nomogram while the validation cohort was used to validate the performance.

Quantitative data were described as mean ± standard deviation (SD) while categorical variables were presented as number and the percentage (*N*, %). The Cox proportional hazards regression was performed to identify the prognostic factors. The primary outcome was overall survival (OS), which was defined as the interval between the diagnosis of BC to all causes of death. The last follow-up date was on July 2020. The nomogram was formulated using the survival package in R. The discriminative ability of the model was evaluated with Harrell's concordance index (C-index) and receiver operating characteristic (ROC). The calibration curves (1000 bootstrap resamples) were performed to evaluate calibration ability of the nomogram.

The IBM SPSS Statistics (version 26.0, Armonk, NY, USA) was used for statistical analyses. The construction of prognostic nomogram and subsequent validation were performed with R version 4.0.0 (R Foundation for Statistical Computing, Vienna, Austria; https://www.r-project.org). All statistical tests were two-sided, and *P* < 0.05 was considered significant.

### 2.4. Ethical Statement

The present study complied with the 1964 Helsinki Declaration and its later amendments or comparable ethical standards, and the Research Ethics Board of the Tianjin Medical University Cancer Institute and Hospital approved the study (bc2021010).

## 3. Results

### 3.1. Demographic and Clinicopathologic Characteristics

A total of 343 patients were eventually selected in the present study. After random grouping of the total cohort, there were 243 and 100 cases in the construction cohort and validation cohort, respectively.

For the total cohort, the median age was 48.0 (interquartile range, IQR: 41.0–57.0) years and patients with age ≤45 years, 46–55 years, and >55 years accounted for 39.1%, 31.8%, and 29.2%, respectively. The majority of patients were married (*N* = 324, 94.5%). Most of the tumors were 2–5 cm (*N* = 175, 51.0%) and with positive nodal status (*N* = 276, 80.5%). The tumor grade was I-II and III in 69.4% (*N* = 238) and 30.6% (*N* = 105) of the patients. The percentages of patients with ER, PR, Her-2, and Ki-67 positive status were 69.1% (*N* = 237), 56.6% (*N* = 194), 21.6% (*N* = 74), and 25.4% (*N* = 87), respectively. Distant metastasis was found among 119 patients in the liver, 36 patients in the brain, 137 patients in the lung, and 76 patients in other organs. More detailed information about demographic and clinicopathologic characteristics of the construction cohort and validation cohort is shown in Table 1.

### 3.2. Synchronous Bone Metastasis and Metachronous Bone Metastasis

**Figure 2:**
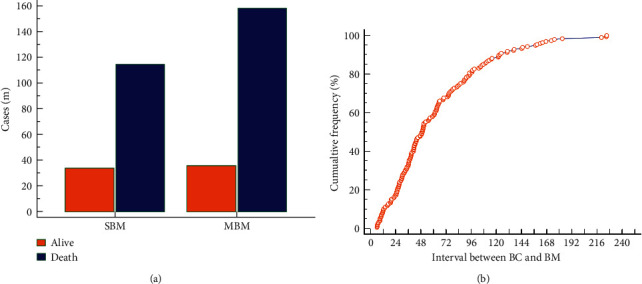
The distribution of survival outcome of synchronous bone metastasis and metachronous bone metastasis (a). The cumulative frequency of bone metastasis in metachronous bone metastasis patients (b).

According to previous definition, there were 148 SBM patients and195 MBM patients. The distribution of survival outcome of SBM and MBM patients is shown in Figure 2(a). For MBM patients, the mean interval between the diagnosis of breast cancer and bone metastasis was 61.9 (6.2–225.2) months. The cumulative frequency is shown in Figure 2(b).

For SBM patients, 95.9% (142/148) presented axial skeleton (including the skull and vertebrae) metastasis while 46.6% (69/148) presented appendicular skeleton metastasis. The number of patients with axial skeleton and appendicular skeleton metastasis were 192 (98.5%) and 98 (50.3%) in MBM patients.

### 3.3. Survival and Prognostic Factors of Breast Cancer Patients with BM

At the last follow-up, a total of 273 patients deceased. The mean OS was 86.3 (95% CI: 77.1–95.6) months and the median OS was 63.2 (95% CI: 52.4–74.0) months. The 1-, 2-, 5-, and 10-year survival rates were 88.9%, 69.3%, 52.8%, and 25.8%, respectively.

In the construction cohort, a total of 190 patients died at the last follow-up. The mean OS and the median OS were 92.8 (95% CI: 80.7–104.9) and 70.0 (95% CI: 59.2–80.8) months, respectively. The 1-, 2-, 5-, and 10-year survival rates were 90.5%, 71.9%, 56.3%, and 27.3%, respectively. The following variables were associated with survival: age at diagnosis, ABO blood type, CA153, ALP, tumor grade, lymph node metastasis, ER status, PR status, Her-2 status, Ki-67 status, surgery for BC, chemotherapy, endocrinotherapy, SBM/MBM, performance of BPs, liver metastasis, lung metastasis, and other organs metastasis. After adjusting all these characteristics in multivariate analysis, elevated ALP (HR = 1.71, 95% CI: 1.16–2.51; *P*=0.006), no surgery for breast cancer (HR = 2.19, 95% CI: 1.30–3.70; *P*=0.003), SBM (HR = 1.98, 95% CI: 1.22–3.22; *P*=0.006), and liver metastasis (HR = 1.68, 95% CI: 1.20–2.37; *P*=0.003) were independent prognostic factors for worse survival. More details about the univariate and multivariate Cox regression analysis are shown in Tables 2 and 3, respectively.

### 3.4. Construction and Validation of Nomogram

**Figure 3:**
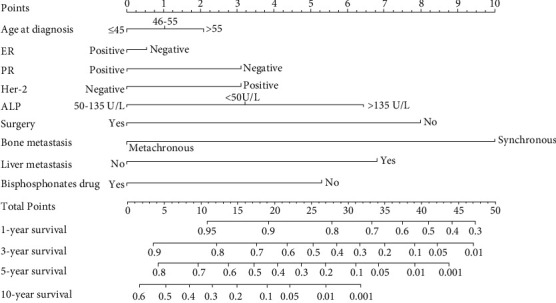
The nomogram to predict survival outcomes for breast cancer patients with bone metastasis.

**Figure 4:**
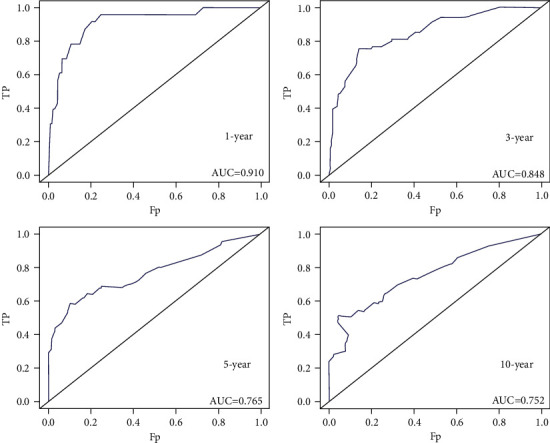
The ROC curves to predict 1-year, 3-year, 5-year, and 10-year survival in construction cohort.

**Figure 5:**
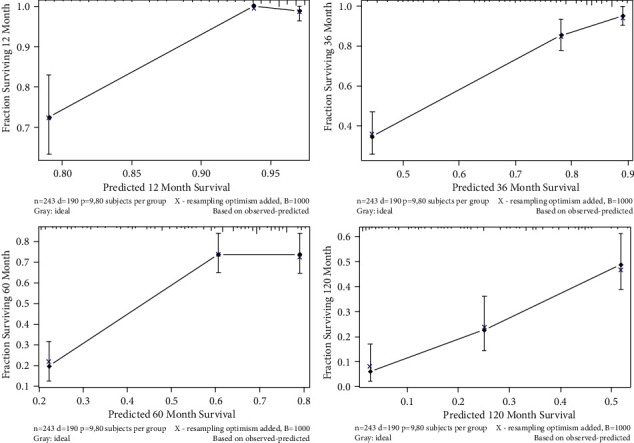
The calibration curves in construction cohort.

As shown in Figure 3, the abovementioned four independent prognostic factors and other five factors (including age at diagnosis, ER status, PR status, Her-2 status, and the performance of BPs) were included to construct a prognostic nomogram. The C-index was 0.714 (95% CI: 0.636–0.792) and the AUC of the nomogram for 1 year, 3 years, 5 years, and 10 years were 0.910, 0.848, 0.765, and 0.752, respectively (Figure 4). The calibration curve revealed good agreement between the predicted and observed probabilities. All the calibration curves were close to the 45-degree line (Figure 5).

**Figure 6:**
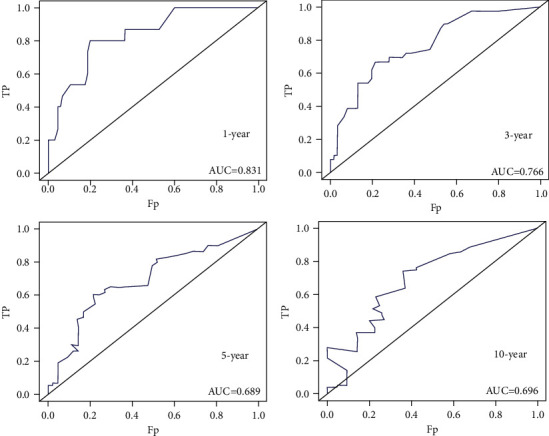
The ROC curves to predict 1-year, 3-year, 5-year, and 10-year survival in validation cohort.

**Figure 7:**
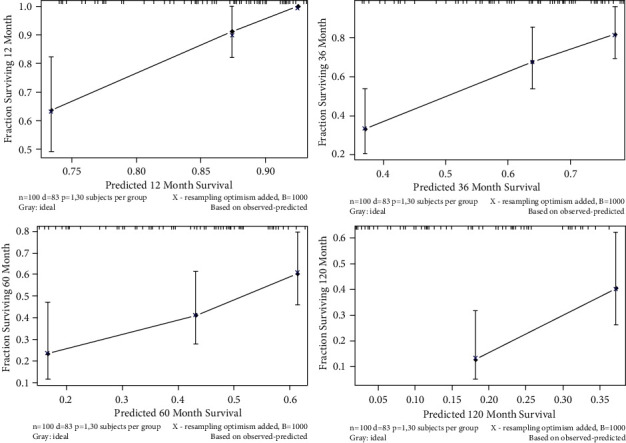
The calibration curves in validation cohort.

In the validation cohort, the nomogram showed satisfactory strength of discrimination. The C-index was 0.705 (95% CI: 0.705) and the AUC for 1 year, 3 years, 5 years, and 10 years were 0.831, 0.766, 0.689, and 0.696, respectively (Figure 6). Excellent ability of calibration was achieved with all calibration curves close to the 45-degree line (Figure 7).

## 4. Discussion

In the current study, a Chinese breast cancer bone metastasis cohort from the Department of Bone and Soft Tissue Tumor was described and associated prognostic factors were identified. Unlike cohorts based on the public dataset, our cohort comprised more treatment information and laboratory data. Besides, SBM and MBM were distinguished according to the time of BM. Previous studies demonstrated the impact of these variables on prognosis prediction and our study further confirmed that [19–23]. Meanwhile, these variables were incorporated to construct a predictive nomogram with satisfactory discrimination and calibration.

As one of the biochemical bone markers, ALP indicated tumor burden and distant metastasis [24]. Consistent with previous studies, elevated ALP was an independent prognostic factor in the present study [23, 25]. Patients with elevated ALP were at 1.71-fold risk of death compared with those with normal ALP. In a retrospective study focusing on bone-only metastasis patients, the median OS were 31.0 and 15.0 months for patients with normal and elevated serum ALP (*P*=0.002) [22]. Better median OS (48.0 months) was suggested among patients with elevated ALP in the present study, which may be attributed to the high proportion of MBM patients.

In recent years, the definition of BM (SBM/MBM) was a global concern in distant metastasis [26, 27]. Sufficient evidence showed the significant difference between SBM and MBM in clinicopathologic characteristics and survival outcome [21, 26, 28]. However, SBM and MBM were still not well defined, which might be the main reason for inconsistent conclusions in previous studies [26, 27]. Although five characteristics (including age at diagnosis, ER status, PR status, Her-2 status, and the performance of BPs) were not independent prognostic factors in the present study, they were integrated in the nomogram after considering their significant effects on prognosis in literatures [6, 29, 30].

Several nomograms were constructed based on SEER database to predict the prognosis of MBC patients at diagnosis [18, 19]. A retrospective study comprising 5,860 BCBM patients reported prognostic nomograms to predict overall survival (OS) and cancer-specific survival (CSS) [19]. The C-index of OS and CSS in the construction cohort was 0.705 and 0.710, respectively. Calibration plots showed the prediction curves were close to 45 degrees in two models [19]. Another SEER-based study enrolled 7,199 stage IV BC patients to predict 1-year and 3-year OS rates, which can stratify patients into different risk groups for clinic demands [18]. These two nomograms were well established and presented good discrimination and calibration. However, due to the limitation of SEER database, the patients with MBM were not involved. Thus, the models cannot be widely used in clinic. At the same time, seldom Chinese patients were recorded; considering racial disparity in cancer, a cohort based on Chinese population is needed.

The present study had some limitations. First, as with many retrospective studies, there was inherent selection bias that cannot be assessed or avoided. Second, missing data were completed by statistical methods, which may lead to the reduction of sample's representativeness. Last but not least, limited Department of Bone and Soft Tissue Tumor was established in China; only internal validation was performed. The generalizability of model should be validated in further studies.

## 5. Conclusions

The current study provided a perspective on prognosis of BCBM. Elevated ALP, no surgery for breast cancer, synchronous bone metastasis, and liver metastasis were four independent prognostic factors for poor survival. A predictive nomogram was constructed and validated. The validation showed good performance. In the Department of Surgery, for the BM patients with potential surgical indication, these findings can be potentially used in survival estimation and individualized treatment planning generation.

## Figures and Tables

**Table 1:** Demographic and clinicopathologic characteristics in the present study.

Subject characteristics	Total cohort	Construction cohort	Validation cohort	*χ* ^2^	*P* value
	*N* (%)	*N* (%)	*N* (%)		
*Age*					
18–45	134 (39.1)	98 (40.3)	36 (36.0)	0.594	*0.743*
46–55	109 (31.8)	75 (30.9)	34 (34.0)		
>55	100 (29.2)	70 (28.8)	30 (30.0)		

*Marital status*					
Married	324 (94.5)	228 (93.8)	96 (96.0)	0.639	*0.424*
Unmarried	19 (5.5)	15 (6.2)	4 (4.0)		

*History of smoking*					
Yes	17 (5.0)	11 (4.5)	6 (6.0)	0.326	*0.568*
No	326 (95.0)	232 (95.5)	94 (94.0)		

*Alcohol consumption*					
Yes	14 (4.1)	12 (4.9)	2 (2.0)	1.562	*0.211*
No	329 (95.9)	231 (95.1)	98 (98.0)		

*Menstrual status*					
Menstruation	108 (31.5)	80 (32.9)	28 (28.0)	0.796	*0.372*
Menopause	235 (68.5)	163 (67.1)	72 (72.0)		

*History of abortion*					
No	236 (68.8)	162 (66.7)	74 (74.0)	1.775	*0.183*
Yes	107 (31.2)	81 (33.3)	26 (26.0)		

*Family history of tumor*					
Yes	83 (24.2)	61 (25.1)	22 (22.0)	0.372	*0.542*
No	260 (75.8)	182 (74.9)	78 (78.0)		

*ABO blood type*					
A type	90 (26.2)	66 (27.2)	24 (24.0)	0.549	*0.908*
B type	116 (33.8)	82 (33.7)	34 (34.0)		
AB type	46 (13.4)	31 (12.8)	15 (15.0)		
O type	91 (26.5)	64 (26.3)	27 (27.0)		

*HGB*					
115–150 g/L	276 (80.5)	194 (79.8)	82 (82.0)	0.227	*0.893*
<115 g/L	59 (17.2)	43 (17.7)	16 (16.0)		
>150 g/L	8 (2.3)	6 (2.5)	2 (2.0)		

*CA153*					
0–25 U/ml	113 (32.9)	75 (30.9)	38 (38.0)	1.633	*0.201*
>25 U/ml	230 (67.1)	168 (69.1)	62 (62.0)		

*CEA*					
0–5 ng/ml	171 (49.9)	116 (47.7)	55 (55.0)	1.495	*0.221*
>5 ng/ml	172 (50.1)	127 (52.3)	45 (45.0)		

*ALP*					
50–135 U/L	227 (66.2)	164 (67.5)	63 (63.0)	0.659	*0.719*
<50 U/L	26 (7.6)	18 (7.4)	8 (8.0)		
>135 U/L	90 (26.2)	61 (25.1)	29 (29.0)		

*Serum calcium*					
2.10–2.55 mmol/L	228 (66.5)	165 (67.9)	63 (63.0)	1.050	*0.592*
<2.10 mmol/L	38 (11.1)	27 (11.1)	11 (11.0)		
>2.55 mmol/L	77 (22.4)	51 (21.0)	26 (26.0)		

*Pathology*					
Ductal carcinoma	219 (63.8)	155 (63.8)	64 (64.0)	0.001	*0.970*
Others	124 (36.2)	88 (36.2)	36 (36.0)		

*Grade*					
I-II	238 (69.4)	172 (70.8)	66 (66.0)	0.763	*0.382*
III	105 (30.6)	71 (29.2)	34 (34.0)		

*Tumor size*					
<2 cm	123 (35.9)	80 (32.9)	43 (43.0)	3.187	*0.203*
2–5 cm	175 (51.0)	129 (53.1)	46 (46.0)		
>5 cm	45 (13.1)	34 (14)	11 (11.0)		

*Lymph node metastasis*					
Yes	276 (80.5)	198 (81.5)	78 (78.0)	0.546	*0.460*
No	67 (19.5)	45 (18.5)	22 (22.0)		

*ER status*					
Positive	237 (69.1)	165 (67.9)	72 (72.0)	0.557	*0.455*
Negative	106 (30.9)	78 (32.1)	28 (28.0)		

*PR status*					
Positive	194 (56.6)	134 (55.1)	60 (60.0)	0.680	*0.410*
Negative	149 (43.4)	109 (44.9)	40 (40.0)		

*Her-2 status*					
Positive	74 (21.6)	51 (21.0)	23 (23.0)	0.170	*0.681*
Negative	269 (78.4)	192 (79.0)	77 (77.0)		

*Ki-67 status*					
Positive	87 (25.4)	60 (24.7)	27 (27.0)	0.199	*0.655*
Negative	256 (74.6)	183 (75.3)	73 (73.0)		

*Surgery for BC*					
Yes	262 (76.4)	186 (76.5)	76 (76.0)	0.012	*0.914*
No	81 (23.6)	57 (23.5)	24 (24.0)		

*Chemotherapy*					
No	21 (6.1)	11 (4.5)	10 (10.0)	3.692	*0.055*
Yes	322 (93.9)	232 (95.5)	90 (90.0)		

*Radiotherapy*					
No	225 (65.6)	160 (65.8)	65 (65.0)	0.022	*0.881*
Yes	118 (34.4)	83 (34.2)	35 (35.0)		

*Endocrinotherapy*					
No	183 (53.4)	129 (53.1)	54 (54.0)	0.024	*0.878*
Yes	160 (46.6)	114 (46.9)	46 (46.0)		

*Targeted therapy*					
No	302 (88.0)	212 (87.2)	90 (90.0)	0.512	*0.474*
Yes	41 (12.0)	31 (12.8)	10 (10.0)		

*Time of BM*					
SBM	148 (43.1)	104 (42.8)	44 (44.0)	0.042	*0.838*
MBM	195 (56.9)	139 (57.2)	56 (56.0)		

*Radiotherapy for BM*					
No	283 (82.5)	202 (83.1)	81 (81.0)	0.222	*0.637*
Yes	60 (17.5)	41 (16.9)	19 (19.0)		

*Pathological fracture*					
No	310 (90.4)	222 (91.4)	88 (88.0)	0.919	*0.338*
Yes	33 (9.6)	21 (8.6)	12 (12.0)		

*Spinal cord compression*					
No	333 (97.1)	236 (97.1)	97 (97.0)	0.004	*0.952*
Yes	10 (2.9)	7 (2.9)	3 (3.0)		

*Surgery for BM*					
No	334 (97.4)	237 (97.5)	97 (97.0)	0.078	*0.780*
Yes	9 (2.6)	6 (2.5)	3 (3.0)		

*Performance of BPs*					
No	84 (24.5)	56 (23.0)	28 (28.0)	0.941	*0.332*
Yes	259 (75.5)	187 (77.0)	72 (72.0)		

*Liver metastasis*					
No	224 (65.3)	155 (63.8)	69 (69.0)	0.850	*0.357*
Yes	119 (34.7)	88 (36.2)	31 (31.0)		

*Brain metastasis*					
No	307 (89.5)	217 (89.3)	90 (90.0)	0.037	*0.848*
Yes	36 (10.5)	26 (10.7)	10 (10.0)		

*Lung metastasis*					
No	206 (60.1)	147 (60.5)	59 (59.0)	0.066	*0.797*
Yes	137 (39.9)	96 (39.5)	41 (41.0)		

*Other organs metastasis*					
No	267 (77.8)	191 (78.6)	76 (76.0)	0.278	*0.598*
Yes	76 (22.2)	52 (21.4)	24 (24.0)		

HGB: hemoglobin; CA153: carbohydrate antigen 153; CEA: carcinoembryonic antigen; ALP: alkaline phosphatase; ER: estrogen receptor; PR: progesterone receptor; Her-2: human epidermal growth factor receptor 2; BC: breast cancer; BM: bone metastasis; SBM: synchronous bone metastasis; MBM: metachronous bone metastasis; BPs: bisphosphonates.

**Table 2:** Univariate Cox proportional hazard regression model for analyzing associated factors in the construction cohort.

Subject characteristics	B	SE	Wald	*P* value	HR	HR 95% CI
Age						
18–45			11.006	0.004	1.00	Ref
46–55	0.169	0.176	0.916	0.338	1.18	0.84–1.67
>55	0.590	0.180	10.675	0.001	1.80	1.27–2.57

Marital status						
Married					1.00	Ref
Unmarried	0.230	0.343	0.449	0.503	1.26	0.64–2.46

History of smoking						
Yes					1.00	Ref
No	0.172	0.364	0.224	0.636	1.19	0.58–2.43

Alcohol consumption						
Yes					1.00	Ref
No	0.182	0.342	0.281	0.596	1.20	0.61–2.35

Menstrual status						
Menstruation					1.00	Ref
Menopause	−0.211	0.161	1.717	0.190	0.81	0.59–1.11

History of abortion						
No					1.00	Ref
Yes	0.010	0.157	0.004	0.952	1.01	0.74–1.38

Family history of tumor						
Yes					1.00	Ref
No	−0.079	0.171	0.212	0.645	0.92	0.66–1.29

ABO blood type						
A type			6.885	0.076	1.00	Ref
B type	0.092	0.188	0.240	0.624	1.10	0.76–1.59
AB type	0.558	0.235	5.656	0.017	1.75	1.10–2.77
O type	−0.002	0.204	0	0.994	1.00	0.67–1.49

HGB						
115–150 g/L			2.906	0.234	1.00	Ref
<115 g/L	0.098	0.193	0.258	0.611	1.10	0.76–1.61
>150 g/L	0.758	0.457	2.758	0.097	2.14	0.87–5.22

CA153						
0–25 U/ml					1.00	Ref
>25 U/ml	−0.342	0.158	4.684	0.030	0.71	0.52–0.97

CEA						
0–5 ng/ml					1.00	Ref
>5 ng/ml	−0.142	0.146	0.946	0.331	0.87	0.65–1.16

ALP						
50–135 U/L			7.375	0.025	1.00	Ref
<50 U/L	0.118	0.267	0.197	0.657	1.13	0.67–1.90
>135 U/L	0.451	0.166	7.373	0.007	1.57	1.13–2.18

Serum calcium						
2.10–2.55 mmol/L			1.613	0.446	1.00	Ref
<2.10 mmol/L	0.185	0.232	0.636	0.425	1.20	0.76–1.89
>2.55 mmol/L	0.207	0.185	1.251	0.263	1.23	0.86–1.77

Pathology						
Ductal carcinoma					1.00	Ref
Others	0.227	0.152	2.240	0.134	1.26	0.93–1.69

Grade						
I-II					1.00	Ref
III	0.470	0.160	8.654	0.003	1.60	1.17–2.19

Tumor size						
<2 cm			0.816	0.665	1.00	Ref
2–5 cm	0.073	0.165	0.196	0.658	1.08	0.78–1.49
>5 cm	0.214	0.238	0.814	0.367	1.24	0.78–1.97

Lymph node metastasis						
Yes					1.00	Ref
No	−0.459	0.191	5.766	0.016	0.63	0.44–0.92

ER status						
Positive					1.00	Ref
Negative	0.330	0.154	4.615	0.032	1.39	1.03–1.88

PR status						
Positive					1.00	Ref
Negative	0.396	0.146	7.390	0.007	1.49	1.12–1.98

Her-2 status						
Positive					1.00	Ref
Negative	−0.411	0.172	5.735	0.017	0.66	0.47–0.93

Ki-67 status						
Positive					1.00	Ref
Negative	0.522	0.176	8.791	0.003	1.69	1.19–2.38

Surgery for BC						
Yes					1.00	Ref
No	1.372	0.181	57.553	<0.001	3.94	2.77–5.62

Chemotherapy						
No					1.00	Ref
Yes	−0.802	0.344	5.430	0.020	0.45	0.23–0.88

Radiotherapy						
No					1.00	Ref
Yes	−0.036	0.150	0.058	0.810	0.97	0.72–1.30

Endocrinotherapy						
No					1.00	Ref
Yes	−0.473	0.147	10.35	0.001	0.62	0.47–0.83

Targeted therapy						
No					1.00	Ref
Yes	0.211	0.211	0.998	0.318	1.24	0.82–1.87

Time of BM						
SBM					1.00	Ref
MBM	−1.104	0.163	45.613	<0.001	0.33	0.24–0.46

Radiotherapy for BM						
No					1.00	Ref
Yes	0.085	0.186	0.208	0.649	1.09	0.76–1.57

Pathological fracture						
No					1.00	Ref
Yes	0.300	0.263	1.304	0.254	1.35	0.81–2.26

Spinal cord compression						
No					1.00	Ref
Yes	−0.066	0.416	0.026	0.873	0.94	0.41–2.11

Surgery for BM						
No					1.00	Ref
Yes	0.077	0.455	0.029	0.865	1.08	0.44–2.63

Performance of BPs						
No					1.00	Ref
Yes	−0.382	0.166	5.288	0.021	0.68	0.49–0.95

Liver metastasis						
No					1.00	Ref
Yes	0.469	0.147	10.140	0.001	1.60	1.2–2.13

Brain metastasis						
No					1.00	Ref
Yes	0.355	0.212	2.807	0.094	1.43	0.94–2.16

Lung metastasis						
No					1.00	Ref
Yes	−0.311	0.150	4.335	0.037	0.73	0.55–0.98

Other organs metastasis						
No					1.00	Ref
Yes	−0.366	0.179	4.182	0.041	0.69	0.49–0.99

HGB: hemoglobin; CA153: carbohydrate antigen 153; CEA: carcinoembryonic antigen; ALP: alkaline phosphatase; ER: estrogen receptor; PR: progesterone receptor; Her-2: human epidermal growth factor receptor 2; BC: breast cancer; BM: bone metastasis; SBM: synchronous bone metastasis; MBM: metachronous bone metastasis; BPs: bisphosphonates.

**Table 3:** Multivariate Cox proportional hazard regression model for analyzing the independent prognostic factors in the construction cohort.

Subject characteristics	B	SE	Wald	*P* value	HR	HR 95% CI
Age						
18–45			0.332	0.847	1.00	Ref
46–55	0.087	0.192	0.206	0.650	1.09	0.75–1.59
>55	−0.023	0.224	0.010	0.918	0.98	0.63–1.52

ABO blood type						
A type			5.191	0.158	1.00	Ref
B type	0.315	0.204	2.376	0.123	1.37	0.92–2.04
AB type	0.495	0.265	3.503	0.061	1.64	0.98–2.76
O type	0.047	0.231	0.042	0.837	1.05	0.67–1.65

CA153						
0–25 U/ml					1.00	Ref
>25 U/ml	−0.268	0.189	2.005	0.157	0.77	0.53–1.11

ALP						
50–135 U/L			7.448	0.024	1.00	Ref
<50 U/L	0.090	0.290	0.097	0.756	1.09	0.62–1.93
>135 U/L	0.536	0.197	7.448	0.006	1.71	1.16–2.51

Grade						
I-II					1.00	Ref
III	0.124	0.197	0.392	0.531	1.13	0.77–1.67

Lymph node metastasis						
Yes					1.00	Ref
No	−0.075	0.209	0.129	0.719	0.93	0.62–1.40

ER status						
Positive					1.00	Ref
Negative	−0.066	0.214	0.094	0.759	0.94	0.62–1.42

PR status						
Positive					1.00	Ref
Negative	0.289	0.191	2.303	0.129	1.34	0.92–1.94

Her-2 status						
Positive					1.00	Ref
Negative	−0.207	0.197	1.106	0.293	0.81	0.55–1.20

Ki-67 status						
Positive					1.00	Ref
Negative	0.341	0.207	2.714	0.099	1.41	0.94–2.11

Surgery for BC						
Yes					1.00	Ref
No	0.786	0.267	8.655	0.003	2.19	1.30–3.70

Chemotherapy						
No					1.00	Ref
Yes	−0.373	0.406	0.845	0.358	0.69	0.31–1.53

Endocrinotherapy						
No					1.00	Ref
Yes	−0.080	0.190	0.178	0.673	0.92	0.64–1.34

Time of BM						
MBM					1.00	Ref
SBM	0.684	0.247	7.646	0.006	1.98	1.22–3.22

Performance of BPs						
No					1.00	Ref
Yes	−0.308	0.200	2.364	0.124	0.74	0.50–1.09

Liver metastasis						
No					1.00	Ref
Yes	0.521	0.175	8.872	0.003	1.68	1.20–2.37

Lung metastasis						
No					1.00	Ref
Yes	−0.216	0.165	1.725	0.189	0.81	0.58–1.11

Other organs metastasis						
No					1.00	Ref
Yes	−0.288	0.197	2.139	0.144	0.75	0.51–1.10

CA153: carbohydrate antigen 153; ALP: alkaline phosphatase; ER: estrogen receptor; PR: progesterone receptor; Her-2: human epidermal growth factor receptor 2; BC: breast cancer; BM: bone metastasis; SBM: synchronous bone metastasis; MBM: metachronous bone metastasis; BPs: bisphosphonates.

## Data Availability

The datasets generated during and/or analysed during the current study are available from the corresponding author on reasonable request.
